# ASIC-based design of NMR system health monitor for mission/safety–critical applications

**DOI:** 10.1186/s40064-016-2249-7

**Published:** 2016-05-16

**Authors:** P. Balasubramanian

**Affiliations:** School of Computer Engineering, Nanyang Technological University, 50 Nanyang Avenue, Singapore, 639798 Singapore

**Keywords:** N-modular redundancy (NMR), Triple modular redundancy (TMR), Quintuple modular redundancy (QMR), Reliability, Fault tolerance, Majority voter, ASIC, CMOS, Standard cells

## Abstract

N-modular redundancy (NMR) is a generic fault tolerance scheme that is widely used in safety–critical circuit/system designs to guarantee the correct operation with enhanced reliability. In passive NMR, at least a majority (N + 1)/2 out of N function modules is expected to operate correctly at any time, where N is odd. Apart from a conventional realization of the NMR system, it would be useful to provide a concurrent indication of the system’s health so that an appropriate remedial action may be initiated depending upon an application’s safety criticality. In this context, this article presents the novel design of a generic NMR system health monitor which features: (i) early fault warning logic, that is activated upon the production of a conflicting result by even one output of any arbitrary function module, and (ii) error signalling logic, which signals an error when the number of faulty function modules unfortunately attains a majority and the system outputs may no more be reliable. Two sample implementations of NMR systems viz. triple modular redundancy and quintuple modular redundancy with the proposed system health monitoring are presented in this work, with a 4-bit ALU used for the function modules. The simulations are performed using a 32/28 nm CMOS process technology.

## Background

Several mission-critical and safety-intensive applications such as space, aerospace, nuclear, power, defence, security, banking and financial, and industrial control and automation incorporate redundancy into their hardware and/or software in order to provide guaranteed correct operation in the face of arbitrary function module fault(s)^1^ (Briere and Traverse 1993; Koren and Mani Krishna 2007; Engelmann et al. 2009; Dubrova 2013). This is because a non-redundant system might turn out to be a single point-of-failure when critical faults get manifested (Johnson 1988). In a passive NMR system constructed using N copies of a function module,^2^ at least (N + 1)/2 of the N function modules, which constitutes a majority, are required to operate correctly in order to guarantee reliable system operation. In any NMR system, the outputs of N identical function modules are combined using majority voting elements and the voters reflect the correct system output through a majority vote. Among the generic NMR systems, triple modular redundancy (TMR) systems which utilize three identical copies of a function module are well-known, popular and highly sought after for the design of safety-intensive applications (Johnson 1988). However, a TMR system can cope with only a single function module fault. Hence in mission-critical space and aerospace systems (Web Reference 1 2001; Azbug and Larrabee 2002), quintuple modular redundancy (QMR) is also used to achieve enhanced reliability. The QMR, which forms a subset of the NMR system, employs five identical copies of a function module and could guarantee fail-safe operation even if any two function modules might fail arbitrarily.

In addition to the successful masking of one (in TMR system) and two (in QMR system) function module faults and still providing the correct system output, it would be useful to concurrently indicate the NMR system’s health to the external environment so that an appropriate remedial action may be initiated to troubleshoot the system in the case of any undesirable corruption. In this context, the word-voter (Mitra and McCluskey 2000), which forms the only relevant work to the best of the author’s knowledge, was proposed exclusively to improve the data integrity of TMR system architectures. It is to be noted that the concept of word-voter is only limited to the TMR system (Mitra and McCluskey 2000), and there are no known mechanisms to ensure data integrity in higher-order (passive) NMR systems. The word-voter would signal an error when more than one function module becomes faulty in a TMR system. The word-voter would not produce any fault indication when just one function module has alone become faulty or when multiple function modules develop disjoint faults^3^ in a TMR system. The error signalling by the word-voter may either be due to the occurrence of temporary function module faults (Lala 1984) in the TMR system from which the system might be able to recover or due to the occurrence of permanent function module faults which would require a full system shutdown and/or urgent repair.

The primary drawback of the word-voter is that it does not provide advance information about fault occurrences which might consequently lead to a sudden, catastrophic failure of the TMR system due to sustained operation and the system has to be forcibly shut down to perform necessary repair or undertake appropriate remedial action when an error is signalled. Given this, supposing fault(s) were detected early during a system’s operation, then there exists a possibility to initiate a prompt remedial action thus pre-empting the likelihood of a potential catastrophic failure through early intervention. Motivated by this observation, this article presents a generic and advanced NMR system health monitoring mechanism that provides an early fault warning signal when even one output of any arbitrary function module in the NMR system produces a conflicting result, thus providing the opportunity to observe and perform an early repair/recovery. Note that error signalling occurs when a majority of the function modules become faulty.

In the rest of this article, ‘‘TMR and QMR—description’’ section describes the fault-tolerant TMR and QMR schemes along with a portrayal of their system reliabilities, their voting elements and their governing equations. In ‘‘Word-voter based TMR and TMR incorporating the proposed system health monitor’’ section, an example TMR system incorporating the word-voter is illustrated, depicting the scenarios when the error signalling tends to be correct and incorrect. This is followed by the specific discussion of an example TMR system which employs the proposed system health monitoring apparatus and is contrasted with the word-voter functionality. In ‘‘NMR implementation with proposed system health monitor’’ section, the realization of a generic NMR system with the proposed system health monitoring mechanism is presented, and its operation is described for the cases of none, single, and multiple faulty function modules. Next, ‘‘Example implementation of TMR and QMR without and with the proposed system health monitor—results and discussion” section presents the simulation results corresponding to a sample implementation of word-voter based TMR, and TMR and QMR systems without and with the proposed system health monitor. The simulation results obtained correspond to a typical case PVT specification of a 32/28 nm CMOS technology. Finally, the conclusions are given in ‘‘Conclusions’’ section.

## TMR and QMR—description

The reliability expressions of simplex (R_Simplex_), TMR (R_TMR_) and QMR (R_QMR_) systems are given by (1), (2) and (3), where R_M_ denotes a function module’s reliability, which signifies the probability of its correct working state. Since identical function modules are deployed in NMR (here, TMR and QMR) systems, their reliabilities may also be treated as equivalent. Under this assumption and simultaneously assuming perfect voting elements, the compact system reliability expressions (1), (2) and (3) are deduced.

1 $${\text{R}}_{\text{Simplex}} = {\text{ R}}_{\text{M}}$$

2 $${\text{R}}_{\text{TMR}} = {\text{ 3R}}_{\text{M}}^{ 2}\, {-}{\text{ 2R}}_{\text{M}}^{ 3}$$

3 $${\text{R}}_{\text{QMR}} = { 1}0{\text{R}}_{\text{M}}^{ 3}\, {-}{\text{ 15R}}_{\text{M}}^{ 4} + {\text{ 6R}}_{\text{M}}^{ 5}$$

**Fig. 1:**
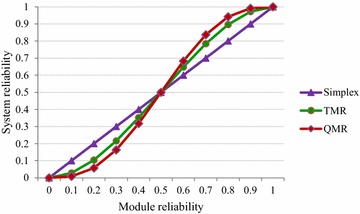
Reliability curves of simplex, TMR and QMR systems plotted as a function of their module reliabilities

A plot of different systems reliabilities versus their module reliabilities is depicted by Fig. 1. Up till R_M_ is equal to 0.5, the simplex system tends to feature better reliability than the TMR or QMR system, but the simplex system does not incorporate any fault tolerance. It may be seen that R_M_ = 0.5 represents the crossover point when the reliabilities of simplex, TMR and QMR systems become equal. For R_M_ ranging from 0.6 to 0.9, the TMR and QMR systems report mean increases in system reliability by 10 and 15.1 % compared to the simplex system. In practice, R_M_ tends to be equal to or greater than 0.9. In fact, when R_M_ = 0.9, the TMR system exhibits enhanced reliability by 8 % over the simplex system and the QMR system reports an improvement in system reliability by 10.2 % for a similar comparison, despite the TMR and QMR systems being fault-tolerant unlike the simplex system which is not fault-tolerant.

**Fig. 2:**
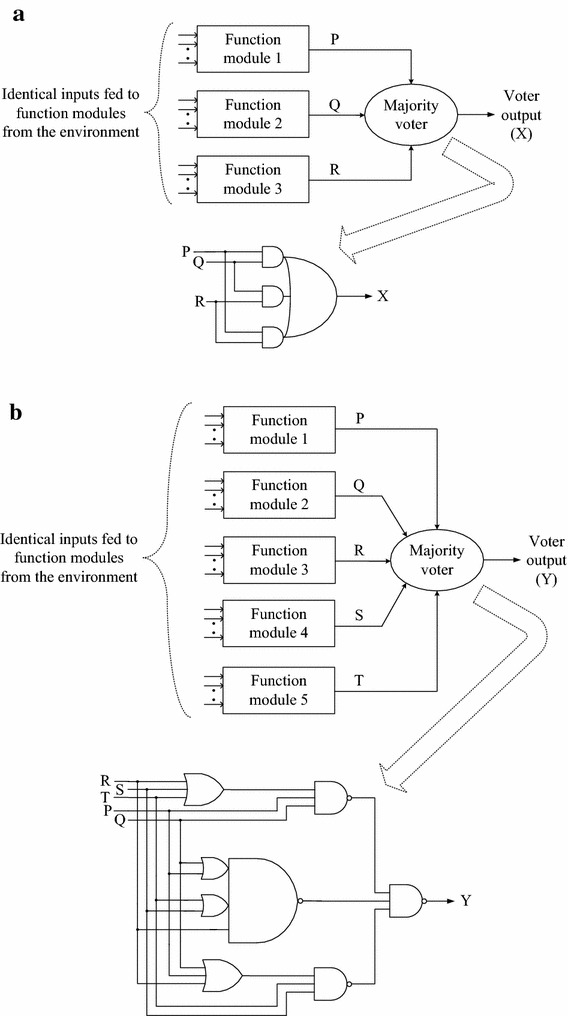
Block diagram of **a** TMR system and **b** QMR system along with the voters

The block schematics of TMR and QMR systems are shown in Fig. 2, portraying the respective gate-level voters. The majority voters corresponding to TMR (Balasubramanian and Mastorakis 2014; Danilov et al. 2014) and QMR (Balasubramanian and Arabnia 2015) are depicted in Figs. 2a and b. The majority voter corresponding to TMR is realized using a single complex gate viz. the AO222 cell, while the majority voter corresponding to QMR is realized using two 3-input OR gates, three 3-input NAND gates, and a complex gate viz. the OAI221 cell. In Fig. 2, P, Q and R represent the outputs of function modules of the TMR system which are equivalent, while, P, Q, R, S and T denote the function module outputs of the QMR system which are also equivalent. In the case of TMR, function modules 1, 2 and 3 are used, while function modules 1 to 5 are employed in the case of QMR. X and Y represent the respective voter/system outputs of TMR and QMR systems. The respective system/voter output equations of TMR and QMR systems are given by (4) and (5). Note that (4) and (5) depict all the Boolean majority clauses of the function module outputs corresponding to TMR and QMR systems. Figures 2a and b synthesize (4) and (5) respectively using standard library cells post logic optimization.

4 $${\text{X }} = {\text{ PQR }} + {\text{ PQ }} + {\text{ QR }} + {\text{ PR}}$$

5 $${\text{Y }} = {\text{ PQRST }} + {\text{ PQRS }} + {\text{ PQRT }} + {\text{ PQST }} + {\text{ PRST }} + {\text{ QRST }} + {\text{ PQR }} + {\text{ PQS }} + {\text{ PQT }} + {\text{ PRS }} + {\text{ PRT }} + {\text{ PST }} + {\text{ QRS }} + {\text{ QRT }} + {\text{ QST }} + {\text{ RST}}$$

## Word-voter based TMR and TMR incorporating the proposed system health monitor

**Fig. 3:**
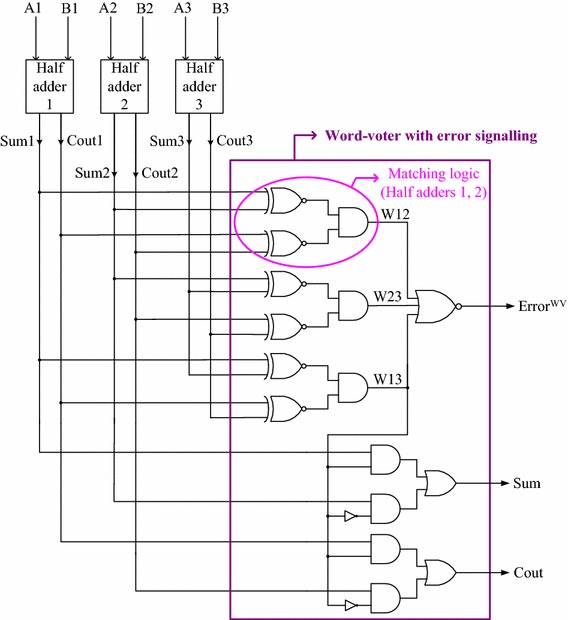
An example word-voter based TMR implementation

The conventional TMR implementation is different from the word-voter based TMR implementation (Mitra and McCluskey 2000) in that the former can handle at most one function module fault, while the latter may be able to selectively handle even multiple function module faults. An example TMR implementation incorporating the word-voter (enclosed within the violet rectangle) is shown in Fig. 3 for an illustration, with the binary half adder used for the function modules. The ‘matching logic’ that confirms whether the outputs of a pair of function modules are equal (signified by binary 1) or not (signified by binary 0) is shown highlighted within the pink oval in Fig. 3.

In Fig. 3, A1 to A3 and B1 to B3 constitute the function modules’ inputs, while Sum1 to Sum3 and Cout1 to Cout3 represent the function modules’ outputs. The respective primary inputs of the half adders’ viz. A1, A2, A3 and B1, B2, B3 are equivalent. The half adders’ corresponding outputs viz. Sum1, Sum2, Sum3 and Cout1, Cout2, Cout3 are also equivalent. Assuming that half adders 1 and 2 are operating correctly and half adder 3 alone has become faulty, Sum1 = Sum2, and Cout1 = Cout2, and hence W12 evaluates to 1. However, Sum 1 ≠ Sum3 and Cout1 ≠ Cout3. Also, Sum2 ≠ Sum3 and Cout2 ≠ Cout3. Thus, W23 = W13 = 0. Since W12 = 1, the error output, Error^WV^ becomes 0, implying that a single faulty function module in the TMR system is successfully masked by the word-voter and it also manages to produce the correct system output by satisfying the Boolean majority, i.e. Sum = Sum2 and Cout = Cout2. Notice that the word-voter will not produce an error signal if a majority of the function modules in the TMR system is maintaining the correct operation.

The word-voter is meant to handle common mode multiple function module faults only in the TMR system. Let us now presume that after the application of specific inputs, the correct outputs of half adder 2 are Sum2 = 1 and Cout2 = 1. Assuming that half adders 1 and 3 have become faulty, let their outputs be assumed as Sum1 = 0, Cout1 = 1; and Sum3 = 1, Cout3 = 0. As a result, the internal word-voter outputs viz. W12, W23 and W13, which govern the matching/non-matching of the pairs of function module outputs will evaluate to 0. Hence, the error output (Error^WV^) produced by the word-voter would be 1, which is correct, indicating that the TMR system is experiencing multiple function module faults, thus suggesting a repair is necessary. Since W13 equals 0, the outputs of half adder 2 viz. Sum2 and Cout2 are reflected as the TMR system outputs i.e. Sum = 1 and Cout = 1, which is also correct.

The only situation which cannot be resolved by the word-voter is the scenario when a majority of function modules in the TMR system become faulty and agree to produce similar incorrect outputs, which would not be signalled as an error because the word-voter would view this as incorrect outputs produced by just one faulty function module. Under the above assumption of the fault-free half adder 2 (i.e. Sum2 = 1 and Cout2 = 1) and faulty half adders 1 and 3, let us now assume that the outputs of half adders 1 and 3 are Sum1 = Sum3 = 0 and Cout1 = Cout3 = 0, instead. Given this, W12 = W23 = 0, but W13 = 1 since the outputs of the faulty half adders 1 and 3 match. As a consequence, Error^WV^ evaluates to 0, indicating no error, which is incorrect. Moreover, since W13 = 1, the outputs of half adder 1 (i.e. Sum1 = Cout1 = 0) are selected and forwarded to the primary outputs viz. Sum and Cout, which is also incorrect.

When any two function modules become faulty at the same time in the TMR system, and if their outputs also match despite being erroneous, then no error may be signalled by the word-voter and the word-voter based TMR system output may also be erroneous, i.e. the word-voter suffers from the problem of data-dependency. However, any generic NMR system would tend to suffer from this limitation as that of the word-voter and this is difficult to deal with at the circuit/system level when passive redundancy is considered. If any two arbitrary function modules become faulty in the TMR system, and provided their respective outputs do not match, correct system output would be produced by the word-voter along with the correct error signalling. However, no advance information about any fault occurrence within the TMR system is signalled to the external environment by the word-voter. This is likely to be a drawback since it prevents the possibility for early fault detection and warning and may not help in carrying out any pro-active system repair if so required.

**Fig. 4:**
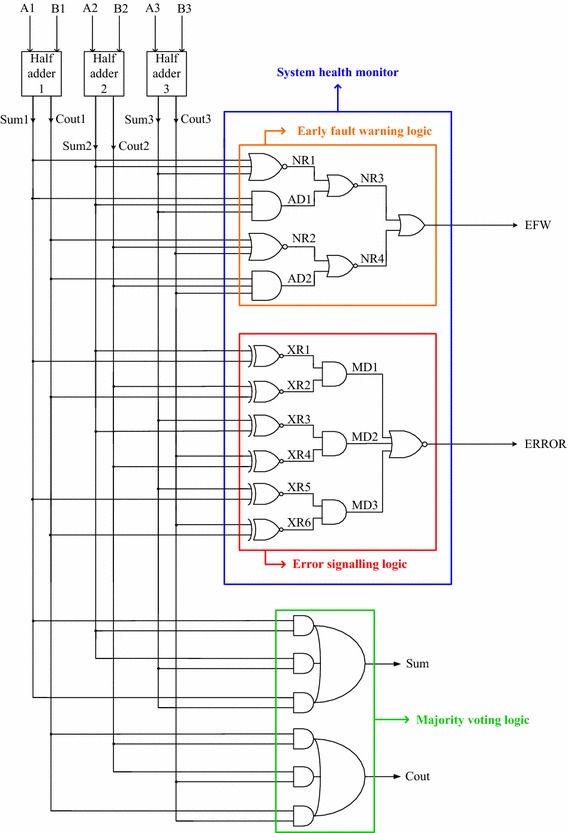
Sample TMR implementation including the proposed system health monitor

To compare and contrast the word-voter based TMR with the TMR implementation featuring the proposed system health monitor, a sample TMR realization with the proposed system health monitoring logic and the majority voting logic is shown in Fig. 4 that utilizes the half adder for the function modules similar to that of Fig. 3. The early fault warning logic, error signalling logic, and the majority voting logic are clearly highlighted in Fig. 4. The early fault warning logic and the error signalling logic constitute the proposed system health monitor. In contrast with the word voter, the proposed system health monitor provides an early fault warning signal even if a single function module output is in disagreement with the rest of its counterparts, and provides an error signal when a majority of the function modules become faulty/fail.

Let us now consider three exemplar scenarios (no function module fault, single function module fault, and multiple function module faults) with respect to Fig. 4 to describe the proposed system health monitoring mechanism. Sum1, Sum2 and Sum3 are the respective sum outputs of half adders 1, 2 and 3; similarly, Cout1, Cout2 and Cout3 form the respective carry outputs of half adders 1, 2 and 3 in Fig. 4. EFW is the output of the early fault warning logic and ERROR is the output of the error signalling logic.

- *No function module fault:* Let us assume that half adders 1, 2 and 3 are fault-free. Hence the respective outputs of the half adders are equivalent, i.e. Sum1 = Sum2 = Sum3 and Cout1 = Cout2 = Cout3. Given this, NR1 = NR2 = 1/0 and AD1 = AD2 = 0/1 respectively. As a result, NR3 = NR4 = 0, which leads to EFW = 0. Also, XR1 up to XR6 would equate to 1, and hence MD1 = MD2 = MD3 = 1, resulting in ERROR = 0. Thus, EFW = ERROR = 0 which reflects the perfect healthy state of the TMR system.
- *Single function module fault:* Let half adders 1 and 2 are fault-free, and half adder 3 is alone faulty. Let Sum1 = Sum2 = Cout1 = Cout2 = 1 and Sum3 = Cout3 = 0. Therefore, in the fault warning logic, NR1 = NR2 = 0; AD1 = AD2 = 0 and NR3 = NR4 = 1, which results in EFW = 1. With regard to the error signalling logic, XR1 = XR2 = 1 and XR3 = XR4 = XR5 = XR6 = 0. Thus MD1 = 1, while MD2 and MD3 are 0 s. Hence, ERROR = 0. The output of the system health monitor is given by EFW = 1 and ERROR = 0, which is indicative of at least one function module fault in the TMR system although the system is said to be operationally healthy, i.e. the TMR system outputs are correct and reliable. The system outputs are Sum = 1 and Cout = 1, since the majority of the function modules’ outputs is 1.
- *Multiple function module faults:* Assume that half adder 1 is alone fault-free, and half adders 2 and 3 have become faulty. Let Sum1 = 1, Cout1 = 0; Sum2 = 1, Cout2 = 1; and Sum3 = 0; Cout3 = 1. Therefore, NR1 = NR2 = AD1 = AD2 = 0; NR3 = NR4 = 1 and hence EFW = 1. In the error signalling logic, XR1 = 1 and XR3 = XR5 = 0 since Sum1 = Sum2 = 1 and Sum3 = 0. Moreover, XR2 = XR6 = 0, while XR4 = 1. Consequently, MD1 = MD2 = MD3 = 0 which results in the issuance of an error signal, viz. ERROR = 1. Thus the system health monitor outputs are EFW = 1 and ERROR = 1, which are correct. The primary system outputs evaluate as Sum = 1 and Cout = 1, which is incorrect since the correct system outputs should have been Sum = Sum1 = 1 and Cout = Cout1 = 0. This shows that when both the fault warning logic and the error signalling logic are activated (i.e. EFW = ERROR = 1), the state of the system health monitor outputs indicates that the system outputs are not correct/reliable.

## NMR implementation with proposed system health monitor

**Fig. 5:**
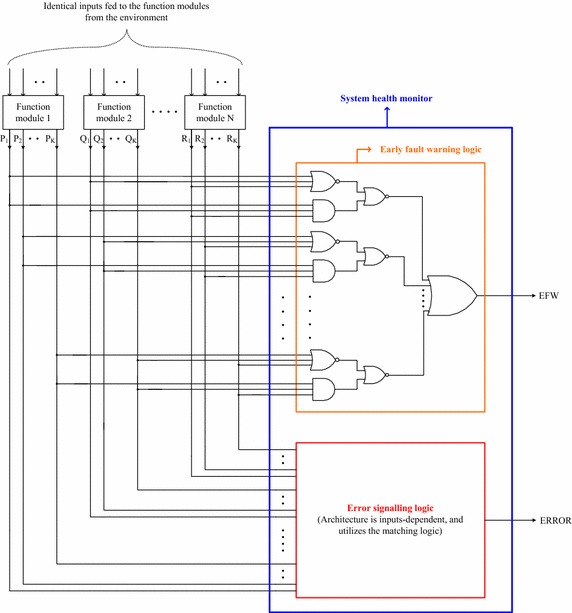
Partial illustration of NMR system with the proposed system health monitor

In this section, a generic NMR system implementation (not just limited to TMR as that of the word-voter) with the proposed health monitoring setup is described. A partial NMR system implementation comprising N identical function modules with each function module producing K outputs is shown in Fig. 5, along with the proposed system health monitor highlighted by the blue rectangle. The voting logic that performs a majority vote on the function modules’ outputs to generate the system outputs, similar to that of Fig. 2, is not shown as part of Fig. 5 as the present focus is on the description of the proposed NMR system health monitor. The proposed system health monitor consists of the early fault warning logic shown enclosed within the orange rectangle, and the error signalling logic which is shown enclosed within the red rectangle in Fig. 5.

The proposed system health monitor produces two outputs: EFW, corresponding to the early fault warning logic; and ERROR, which corresponds to the error signalling logic. Let us first discuss the operation of the fault warning logic, followed by a discussion of the error signalling logic.

In Fig. 5, P_1_ to P_K_, Q_1_ to Q_K_ and R_1_ to R_K_ denote the primary system outputs. It can be seen that the corresponding outputs of all the function modules (for example, P_1_, Q_1_ and R_1_) are given as inputs to both a NOR gate and a AND gate present in the first level of the proposed fault warning logic, whose outputs are combined by NOR gates in the second level and their output is fed to the final-stage OR gate that produces the early fault warning output, EFW. In any arbitrary NMR system featuring N × K outputs, K numbers of N-input NOR gates and AND gates in the first level, K numbers of K-input NOR gates in the second level, and a final K-input OR gate are required to realize the proposed fault warning logic. The gates present at any logic level may be optimally decomposed taking into account the fan-in restrictions of a digital cell library.

Since the function module outputs are simultaneously fed to 3-input NOR and AND gates present in the first level of the fault warning logic, if these gate inputs are 1, the NOR gate will output 0, and the AND gate will output 1. On the contrary, if the gate inputs are 0 s, the NOR gate will output 1, and the AND gate will output 0, i.e. the outputs of NOR and AND gates are mutually exclusive if the applied inputs are the same. In contrast, if the inputs are different, i.e. if one of the inputs is 1 and at least another input is 0, both the NOR and AND gates will output 0. As a result the outputs of the NOR gates present in the second logic level will be 1, leading to an early warning signal issued by the fault warning logic. From the preceding discussions, it may be evident that the proposed fault warning logic is highly sensitive and robust since even a single faulty output of any function module would be promptly detected by the fault warning logic indicating potential fault(s) occurrence in one or more function module(s) comprising the system. In fact, the prompt production of a fault warning signal gives an opportunity for a human monitor to initiate immediate remedial action depending upon the application. It may be noted that this manner of production of an early fault warning signal is absent in the word-voter even for the TMR system configuration.

The primary system outputs viz. P_1_ to P_K_, Q_1_ to Q_K_ and R_1_ to R_K_ are also simultaneously processed by the error signalling logic to generate the ERROR signal. The architecture of the error signalling logic is dependent on the number of function modules outputs and basically utilizes the matching logic (shown in Fig. 3). The purpose of the error signalling logic is to confirm whether or not the respective outputs of a majority group of the function modules match, and if a match is established with respect to even a single majority group of the function modules, the output of the error signalling logic viz. ERROR would be asserted low (i.e. binary 0). Otherwise, ERROR would be asserted high (i.e. binary 1) indicating that the Boolean majority condition of the function modules is violated. An NMR system, where a majority M out of N function modules is expected to operate correctly, would have a total of NC_M_ majority groups, i.e. NC_M_ unique combinations of correctly operating function modules.

**Fig. 6:**
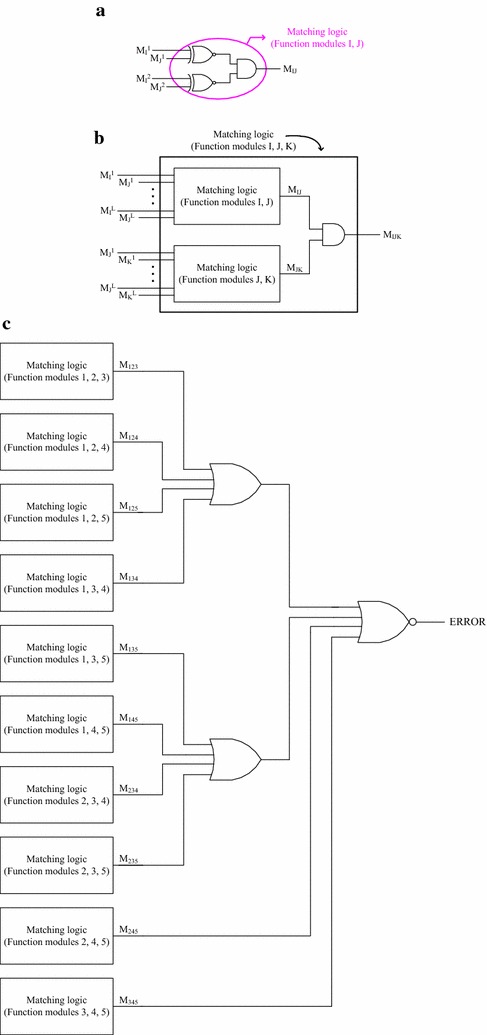
Error signalling logic of a sample QMR system with 5 identical function modules. **a** Matching logic corresponding to 2 function modules, **b** matching logic for 3 function modules and **c** error signalling circuit based on the outputs of matching logic of a QMR system

For illustration, the error signalling logic of a QMR system is depicted in Fig. 6. In Fig. 6a, two function modules I and J are considered, whose respective outputs are M_I_^1^, M_I_^2^ and M_J_^1^, M_J_^2^. The corresponding pairs of outputs viz. (M_I_^1^, M_J_^1^) and (M_I_^2^, M_J_^2^) are given as inputs to two 2-input XNOR gates, which produce the output of 1 if M_I_^1^ = M_J_^1^ and M_I_^2^ = M_J_^2^.

The outputs of the XNOR gates are combined by an AND gate, whose output M_IJ_ is 1 if the two equality conditions (M_I_^1^ = M_J_^1^ and M_I_^2^ = M_J_^2^) are met, and 0 even if one equality condition is not met. Figure 6b shows how the matching logic corresponding to the 3 function modules viz. I, J and K is realized. The matching logic outputs of the pairs of function modules considered (i.e. I, J and J, K) viz. M_IJ_ and M_JK_ are combined using an AND gate to produce the matching logic output (M_IJK_) corresponding to the 3 example function modules.

Figure 6c shows how the error signalling logic of the QMR system is realized. Since 3 out of 5 function modules forms a majority in the QMR system and since a majority of the function modules are expected to be in correct operation, there are a total of 5C_3_ (i.e. 10) combinations reflecting the distinct majority groups of function modules. Although groups of 4 or 5 function modules also form a majority with respect to a QMR system, nevertheless, they would be implicitly covered by the majority groups comprising just 3 function modules. The matching logic outputs of the majority groups are combined using a NOR gate, whose output is designated as ERROR in Fig. 6c. At least one matching logic output corresponding to a majority group of the function modules has to be 1, which would indicate no error. Otherwise, an error signal would be produced conveying that the Boolean majority criterion is violated. The error signalling logic of an NMR system would signal an error if a majority of the function modules becomes faulty, i.e. if only a minority of the function modules exhibit correct operation. The cause of multiple function module failures may be temporary or permanent.

Given that the proposed system health monitor produces two outputs EFW and ERROR, it is important to note that the healthy state of the NMR system is indicated by EFW = 0 and ERROR = 0. In fact, this combination implies that the NMR system health is perfect. When a majority of the function modules outputs are correct, the NMR system health monitor produces EFW = 1 and ERROR = 0. This combination implies that the NMR system is healthy whilst suggesting the need for a potential remedial action since EFW = 1. EFW = 1 implies that the output(s) of single or multiple function modules may have become corrupt; however since the Boolean majority condition is upheld, ERROR = 0. When a majority of the function modules become faulty and only a minority of the function modules outputs agree, the NMR system health monitor outputs EFW = 1 and ERROR = 1. This is reflective of the critical (unhealthy) state of the system and the system outputs may no more be guaranteed to be correct. If the values of EFW and ERROR are maintained as 1 over successive operation cycles, then a permanent system error is said to have occurred, but if the values of EFW and ERROR tend to change over subsequent operating cycles, then it is indicative of a temporary system error. The implications of EFW and ERROR outputs in terms of their binary states for an NMR system incorporating the proposed system health monitor are succinctly portrayed through Table 1.

**Table 1:** Proposed NMR system health monitor outputs and their states interpretation

EFW	ERROR	NMR system health
0	0	Perfectly healthy (no fault)
0	1	Indeterminate (invalid)
1	0	Healthy (with fault masking)
1	1	Unhealthy (unreliable)

The provision of two system health monitor signals viz. EFW and ERROR and that too for a generic NMR system in contrast with just one ERROR signal of the word-voter (and that too only for the TMR system) is more beneficial with regard to suggesting/taking early pre-emptive action to troubleshoot the system faults in any mission/safety–critical NMR system, and this is a major contribution of this article.

## Example implementation of TMR and QMR without and with the proposed system health monitor—results and discussion

A sample implementation of word-voter based TMR, and TMR and QMR systems without and with the proposed system health monitor has been considered, with a 4-bit ALU (Web Reference 2 1988) used for the function modules. The ALU comprises 14 primary inputs and features 8 primary outputs, and is realized using the elements of a digital standard cell library (Synopsys Inc. 2012). The 4-bit ALU consumes 150.45 µm^2^ of Silicon, while the voters corresponding to TMR and QMR systems shown in Figs. 2a and b consume 3.3 and 13.47 µm^2^ of Silicon respectively.

The simulation results viz. power, delay, and area obtained for the sample implementation of TMR, word-voter based TMR (TMR_WV), TMR with system health monitor (TMR_SHM), QMR, and QMR with system health monitor (QMR_SHM) are shown in Table 2. Minimum sized gates were used for all the NMR systems realizations, and the associated wire loads were selected automatically. The structural integrity of different NMR systems and their associated voters without/with the proposed system health monitor were preserved during technology mapping and whilst performing the simulations. This paves the way for a legitimate comparison of the design attributes of different NMR systems being considered in this work. For accurate estimation of the average power dissipation through time-based power analysis, the 4-bit ALU used in all the redundant implementations were supplied with all possible distinct inputs viz. 2^14^ (16,384) input vectors. The input vectors additionally serve as inputs for testing and faults analysis. All the input vectors were supplied at time intervals of 2.5 ns (400 MHz) through test benches. Functional simulations were performed using Synopsys VCS and the functionality of different NMR systems were verified. The.vcd files generated were subsequently used for accurate average power estimation using Synopsys PrimeTime by invoking the time-based power analysis mode. The area and critical path delay metrics were also estimated and are given in Table 2. All the system outputs possess fanout-of-4 drive strength.

**Table 2:** Power, delay, area, and FOM of word-voter based TMR, and various TMR and QMR realizations without and with the proposed system health monitor, considering a 4-bit ALU for the function modules

System configuration	Power (µW)	Delay (ns)	Area (µm^2^)	FOM × 10^6^
TMR	38.33	0.75	477.79	72.81
TMR_WV	46.06	1.01	605.62	35.49
TMR_SHM	52.30	1.03	675.77	27.47
QMR	69.86	0.82	860.02	20.30
QMR_SHM	106	1.25	1380.51	5.47

**Fig. 7:**
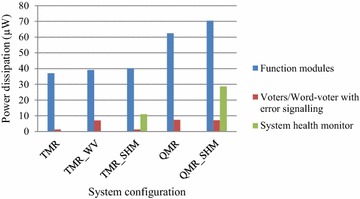
Split-up of power dissipation of function modules, majority voters/word-voter, and system health monitor (if any) corresponding to various TMR and QMR systems

To comprehensively comment on the design parameters, a figure-of-merit (FOM) is defined as the inverse of the product of power, delay and area (PDAP^−1^). Since minimization of power, delay, and area is desirable, a lower PDAP value and thus a higher FOM value are desirable, and the calculated FOM values are given in Table 2. Also, the split-up of average power dissipated by the function modules, voters/word-voter with error signalling, and the proposed system health monitor (where applicable) is portrayed in Fig. 7.

From Table 2, it can be seen that the conventional TMR and QMR systems feature high FOM than the other TMR and QMR system implementations. This is expected because the basic TMR and QMR systems feature only the function modules and the majority voters, while TMR_WV, TMR_SHM and QMR_SHM incorporate extra error signalling logic or the proposed system health monitor. More logic implies more area and consequently more power dissipation, and is found to be the cause for more propagation delay as well. Compared to TMR_WV, the proposed TMR_SHM exhibits a 22.6 % reduction in FOM; nevertheless, the proposed system health monitor is more advanced, robust, and can provide an early fault warning signal compared to the TMR_WV which embeds only the error signalling logic. Also, the proposed system health monitor is generic and can be tailored to suit any NMR system. In comparison with the basic QMR system, the QMR_SHM reports less FOM by 2.7×.

Referring to the chart shown in Fig. 7, it can be seen that the function modules dissipate extra power in the case of TMR_WV, TMR_SHM and QMR_SHM compared to those of their traditional TMR and QMR system counterparts. This is because in case of the latter, the function modules outputs are solely processed by the majority voters, while in the case of the former, the function modules outputs are additionally processed by the error signalling logic or the system health monitor and hence extra power is dissipated. Of the total power dissipated by the TMR_SHM, the proportion of power dissipated by the proposed system health monitor is 20.8 %. In the case of QMR_SHM, this proportion is found to be 27 %.

In general, fault tolerance and fault detection cannot be achieved without introducing redundant and/or extra logic and without involving a trade-off of the design metrics, and quite obviously improvising the fault/failure detection and reporting mechanism i.e. through a system health monitor as discussed in this work would entail an additional trade-off in terms of the design metrics. In mission/safety–critical systems, design metrics trade-off does not form an issue since early fault detection and signalling is more important to initiate an appropriate remedial action so as to ensure the correct and reliable system operation over the scheduled life-time. This is because sudden and catastrophic system fault(s) which might have been prevented through an early intervention following an early system health indication may lead to an unexpected mission-failure or an early aborting of the mission operation which does not augur well for the mission success.

## Conclusions

This article has presented a novel, generic system health monitor for ASIC-based realization of mission/safety–critical NMR systems that gives an early fault warning signal upon the detection of even a single erroneous output by any function module constituting the NMR system, and the signalling of an error once the majority of the function modules in the NMR system become faulty. The provision of a fault warning output serves as an early indicator of at least a single fault occurrence within the NMR system, thus promptly suggesting the need for a likely corrective course of action depending upon an application’s safety criticality. Also, the trade-off involved in the provision of system health indication vis-à-vis the design metrics has been analysed for example TMR and QMR systems implementations.

## Footnotes

1 The term ‘fault’ may refer to single or multiple faults occurring within the function module which may not cause a catastrophic failure of the function module. On the other hand, the term ‘fault’ when used in the context of a function module, as used in this footnote, may mean a complete function module failure implying that the function module outputs are no more reliable. The meaning of the term ‘fault’ therefore has to be carefully interpreted in reference to the context of its usage.

2 The term ‘function module’ generically refers to any circuit/system in this article.

3 Disjoint faults occurring between the function modules of an NMR system are those which do not affect the actual NMR system output(s) due to the absence of any common-mode effect.
